# Language development in children from a public cochlear implant program

**DOI:** 10.1016/j.bjorl.2024.101458

**Published:** 2024-06-17

**Authors:** Alice Lang Silva, Isadora Martins da Silva Stumpf, Laura Prolla Lacroix, Debora Milena Ferreira Alves, Adriana Laybauer da Silveira, Sady Selaimen da Costa, Letícia Petersen Schmidt Rosito

**Affiliations:** a Universidade Federal do Rio Grande do Sul (UFRGS), Programa de Pós-Graduação em Saúde da Criança e do Adolescente, Porto Alegre, RS, Brazil; b Hospital de Clínicas de Porto Alegre (HCPA), Serviço de Otorrinolaringologia, Porto Alegre, RS, Brazil; c Universidade Federal do Rio Grande do Sul (UFRGS), Faculdade de Medicina, Porto Alegre, RS, Brazil; d Hospital de Clínicas de Porto Alegre (HCPA), Serviço de Fonoaudiologia, Porto Alegre, RS, Brazil

**Keywords:** Cochlear implant, Prelingual deafness, Language development disorders

## Abstract

**Objective:**

To evaluate the rate of loss to follow-up in a cochlear implant program from the public health system in Southern Brazil as well as the characteristics of hearing loss, sociodemographic, sociocultural and the development of oral language in children with prelingual deafness.

**Methods:**

Retrospective cohort study with children who underwent CI surgery between 2010 and 2020. Data was collected through of interviews and review of medical records. The language development assessment was performed using the MUSS, MAIS and IT-MAIS scales. For the classification of language development, we used as parameters the values (mean ± SD) found in a previous national study. From those values, the Z-score for each patient at each hearing age (time of experience with the cochlear implant) was calculated.

**Results:**

Of the 225 children implanted between 2010–2020, 129 were included in this study. The rate of loss to follow-up in the program was 42.6%. The mean age at first surgery was 40.5 (±16.9) months, with 77.5% of patients having received a unilateral implant. Language results below the expected for hearing age (<Z-score-1) for the MAIS score were found in 59.7% of the sample, while for the MUSS score the proportion was 62%.

**Conclusions:**

Most patients had an elevated mean age at cochlear implantation and there was a high rate of loss to follow-up and low attendance to speech and programming sessions. An overall poor language performance was found for this pediatric cochlear implant program from the public health system in Southern Brazil.

**Level of evidence:**

Level 3 (Non-randomized cohort study).

## Highlights

• Language performance in public CI program in Southern Brazil is poor.
• Socioeconomic status in the public health system is homogeneous.
• Mean age at cochlear implantation and loss to follow-up are high.

## Introduction

Deafness is the most common congenital sensory deficiency with an incidence of 1–3 per 1000 in healthy neonates and 2–4 per 100 in high-risk infants.^1^, ^2^, ^3^, ^4^, ^5^, ^6^, ^7^ It results in a major impact since it interferes in the processes of oral language development. The limited access to oral communication, on its own, requires numerous adaptations in social and family relations.^5^, ^7^, ^8^, ^9^

Cochlear Implants (CI) provide hearing capacity for patients with profound hearing loss. Nevertheless, the gap for language acquisition between implanted children and their normal hearing peers is still a matter of research around the world.^10^, ^11^, ^12^, ^13^, ^14^, ^15^

Many factors have been implicated in language development outcomes for children with CI, reflecting the uniqueness of each child and their background. Nevertheless, some variables seem the most important: age at implantation,^10^, ^16^, ^17^, ^18^, ^19^, ^20^ family involvement,^21^, ^22^, ^23^, ^24^, ^25^, ^26^ social determinants of health^10^, ^18^, ^23^, ^25^, ^26^, ^27^ and comorbidities.^14^, ^24^, ^28^, ^29^, ^30^, ^31^, ^32^, ^33^, ^34^, ^35^, ^36^

In Brazil, the Public Health System, known as SUS (Sistema Único De Saúde), pays for the whole treatment for patients with profound hearing loss. In practice, we observe limitations of the service offered, especially in postoperative follow-up, but also difficulties of families in maintaining consistency in the demanding path of postoperative rehabilitation.

Therefore, the objectives of our study are to describe the medical, sociodemographic, sociocultural characteristics (profile); to evaluate the access to postoperative rehabilitation (engagement); and to determine the results in language acquisition of pediatric patients from a public cochlear implant program (performance). We called this a PEP strategy.

## Methods

### Study design

This was a retrospective cohort study.

### Subjects

Our sample included all implanted children with prelingual deafness from 2010 to 2020 who fulfilled the following criteria: 1) Maintaining regular appointments in the cochlear implant program (at least one annual appointment); 2) Have at least one audiometry with a cochlear implant in the last 24 months prior to the assessment; 3) Consent from parents or legal guardians to participate in the study. No exclusion criteria were used.

The etiology of deafness was defined after thorough medical history and physical examination, complete audiology tests, imaging exams; genetic testing for connexin 26 mutation and expert assessment (when patients with a set of suggestive changes of syndromes are referred for evaluation in the medical genetics service), prior to the definition of type of treatment. Auditory Neuropathy Spectrum Disorder (ANSD) diagnosis was made through altered responses in the ABR with presence of cochlear microphonics and normal OAEs. All included patients had profound hearing loss prior to CI surgery (assessed using the frequency-specific or steady-state auditory brainstem response, which was absent at 90 dB normal Hearing Level [HL] in all cases, and/or the Visual Reinforcement Audiometry [VRA] using insert earphones).

### Measure of receptive and expressive language development

Each child was evaluated for auditory and speech skills after during one of their regular appointments (or by telephone if there were COVID-19 restrictions) between the years of 2021 and 2022. Children’s skills were evaluated using the Infant Toddler Meaningful Auditory Inventory Scale (IT-MAIS)^37^ for children under 4 years old, Meaningful Auditory Inventory Scale (MAIS)^38^ for children from the age of four, and the Meaningful Use of Speech Scale (MUSS)^39^ for all children. They are parent reports that investigate children’s spontaneous listening behaviors (IT-MAIS and MAIS) and assess children's verbal output capacity (MUSS) in everyday situations.

For the classification of language development (satisfactory or unsatisfactory for the time of experience with a CI), we used as parameters the values (mean and Standard Deviation – SD) found in the study by Comerlatto.^40^ This study has set the clinical benchmark of development for the studied scales at different hearing ages (time of experience with the CI) in a sample of Brazilian children implanted before 36 months, without comorbidities, surgery complications or inner ear malformations. In their research, only the IT-MAIS scale was used, since it’s recommended to assess children aged four years and below. In our study, we also used the IT-MAIS for children younger but preferred to use the MAIS scale to assess children older than 4 years old. Since their great similarity, those results were clustered for the analysis.

A subanalysis of answers for question number 8 of the MUSS score was performed. We considered those answers of particular interest since it evaluates how frequently other adults (not familiar with the child) can understand what they’re saying.

### Medical history, sociodemographic and sociocultural characteristics

Past medical history, sociodemographic and sociocultural data were collected through review of medical records and interviews with the parents. The following variables were evaluated by reviewing the electronic medical record:

• Data on CI surgery: age at the time of surgery; surgical technique; complications; inner ear malformation; lateral or bilateral implant; simultaneous or sequential bilateral implantation.
• Audiometry for evaluation of pure-tone average for thresholds at 500, 1000, 2000 and 4000 Hz. For patients who hadn’t performed any audiometry after 2019, it was offered to perform one during the day of the interview with the audiologist of the program.
• Number of scheduled programming sessions and the number of sessions actually performed since cochlear implant activation, per year.

The following variables were evaluated through an interview with the parents and a review of the child's chart:

• Pregnancy history: prenatal care (number of appointments); STORCH infections encompassing syphilis, *Toxoplasma gondii*, rubella, cytomegalovirus, herpes simplex, and others human immunodeficiency virus; history of depression.
• Neonatal period: type of delivery; Apgar score at 1 and 5 min; need of neonatal intensive care (yes or no); mechanical ventilation; infections (STORCH infections or neonatal sepsis); use of antibiotics; exchange transfusion for jaundice; head and neck malformations; performance of hearing screening and its method.
• History of deafness: family history of deafness; etiology.
• Sociodemographic and sociocultural data: family’s socioeconomic status (defined by number of minimum wages per family: <1, 2–4 and >4); family bilinguism (either sign language or other languages, whether present or not and only if the child had a bilingual family member living in the same house); maternal education (divided by middle school, high school or university level); exposure to screens (either television, computer, smartphone or tablets, measured in number of hours per day); reading habit (never, once a week, once a month or everyday); speech therapy time (whether patient has never had regular speech therapy, or had periods of regular sessions but periods without, or if have always maintained regular sessions since activation) and frequency (never, once a month, once a week or twice a week).

### Statistical analysis

Study data were collected and managed using REDCap (Research Electronic Data Capture) electronic data capture tools hosted at our Hospital. Categorical variables were described as percentages, while continuous variables with normal distribution were described as means with the respective standard deviation and those with asymmetric distribution as medians and interquartile range.

For the classification of language development, we used as parameters the values (mean and Standard Deviation – SD) found in the study by Comerlatto.^40^ From those values, the Z-score for each patient at each hearing age (time of experience with the CI) was calculated. Unsatisfactory results for the MAIS and MUSS score were considered a value below Z-score-1. The SPSS program, version 23.0, was used for the statistical analysis (IBM SPSS Statistics for Windows, Armonk, NY, USA).

### Ethical considerations

All parents or legal guardians signed an informed consent. The study design and subject recruitment were performed according to local ethical requirements. The children were routinely followed at the CI program and had no need for extra appointments to participate in the study nor loss of any kind of regular care.

## Results

A total of 129 children were included in this study but hospital records review showed that 189 children were implanted in the years of 2010–2020. Considering the PEP strategy, our findings concerning patients’ profile, such as mean age at first CI surgery, stimulation modalities, surgical techniques and etiologies of hearing loss from the sample are summarized in Table 1. Twenty-nine children had bilateral CIs and the devices were implanted simultaneously in 21 subjects. Ninety seven percent of the sample belonged to the same socioeconomic status and 96.3% of mothers had prenatal care. Other sociodemographic and sociocultural characteristics that have previously been associated with language development are described in Table 2.

**Table 1:** Characteristics of the sample.

Characteristic	Value
Gender, n (%)	
Female	68 (52.7)
Male	61 (47.3)
Gestational age (weeks), mean (SD); range	37.4 (3.2); 24‒43
Birth weight (g), mean (SD); range	3043 (696); 900‒5355
NICU admission, n (%)	25 (19.4)
Apgar score at 5-min	
<4, no. (%)	2 (2.2)
5‒7, n (%)	6 (6.5)
8‒10, n (%)	86 (91.5)
Stimulation modality, n (%)	
Bilateral	29 (22.5)
Unilateral	80 (62.0)
Bimodal stimulation	20 (15.5)
IC use, hours daily, mean (SD); range	10.3 (4.4); 0‒18
Age at first CI, mo, mean (SD); range	
General	40.5 (16.9); 9‒100
Bilateral	34.7 (19.4); 9‒100
Unilateral	42.1 (15.9); 14‒86
Surgical technique, n (%)	
Round window	28 (21.7)
Cochleostomy	101 (78.3)
Surgical complications, n (%)	2 (1.6)
Reintervention, n (%)	3 (2.3)
Etiology	
Auditory neuropathy	4 (3.1)
Congenital infection	4 (3.1)
Inner ear malformation	8 (6.2)
Genetic syndromic	8 (6.2)
Meningitis	10 (7.8)
Neonatal conditions	13 (10.1)
Genetic nonsyndromic	13 (10.1)
Unknown	69 (53.5)

NICU, Neonatal Intensive Care Unit.

**Table 2:** Profile (sociodemographic/cultural) characteristics.

Characteristic	Value
Family income, n (%)	
<1 minimum wage	8 (6.3)
2–4 minimum wages	115 (90.6)
>4 minimun wages	4 (3.1)
Maternal education, n (%)	
Middle school	35 (27.1)
High school	57 (44.2)
College/university	35 (27.1)
Schooling type, n (%)	
Mainstream	90 (69.8)
Special needs	35 (27.1)
Does not attend	4 (3.1)
Reading habit, n (%)	
Never	56 (43.4)
Once a month	5 (3.9)
Once a week	39 (30.2)
Daily	29 (22.5)
Bilingual family, n (%)	
Yes	37 (28.7)
No	90 (69.8)
Exposure to screens (hours/daily), n (%)	
0–1	7 (5.4)
1–2	18 (14.0)
2–3	29 (22.5)
3–4	26 (20.2)
>4	46 (35.7)
Maternal diagnosis of depression, n (%)	10 (7.8)
Mother’s parity, no, mean (SD)	2.1 (1.2)

Regarding engagement to postoperative rehabilitation, a detailed description can be found in Table 3. Another important aspect related to engagement to treatment was rate of patients lost to follow-up. The children not included in the study were the ones who did not attend any consultations during the years 2021 and 2022 (when the interviews were carried out). That leaves a proportion of 31.7 % of patients who are considered to have abandoned the program (60 out of 189 children). From 22 children, especially the ones implanted until 2015, no contact was possible because the telephones were out of date. With the remaining 38 families, contact was made but they were not included either because they didn’t attend the scheduled appointment (24) or because they had given up on CI usage and chose to use sign language only (14).

**Table 3:** Postoperative follow-up data.

Characteristic	Value
Pure tone average (PTA), mean (SD)	32.6 (18.6)
PTA < 30 dB, (%)	54.3
PTA 30–40 dB, (%)	31
PTA > 40 dB, (%)	14.7
Programming sessions, per year since activation, mean (SD)	
Planned	3.4 (1.8)
Performed	2.4 (1.4)
Speech therapy time, n (%)	
Never	16 (13.2)
Intermittently	16 (13.2)
Always since activation	89 (73.6)
Speech therapy frequency, n (%)	
Never	24 (18.8)
Once a month	10 (7.9)
Once a week	76 (59.0)
Twice a week	16 (12.3)
IC usage, n (%)	
Not using	8 (6.2)
1‒8 h/day	15 (12.5)
9‒14 h/day	64 (49.6)
15‒18 h/day	5 (2.9)

**Figure 1:**
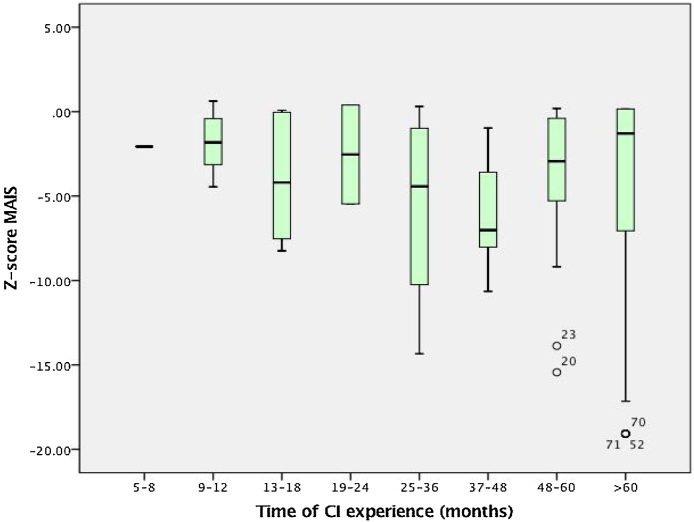
Sample performance on MAIS and IT-MAIS in different hearing ages (time of experience with Cochlear Implant [CI]) in median values with interquartile ranges and vertical bars which denote minimum and maximum values — outliers are depicted by circles outside the bars.

**Figure 2:**
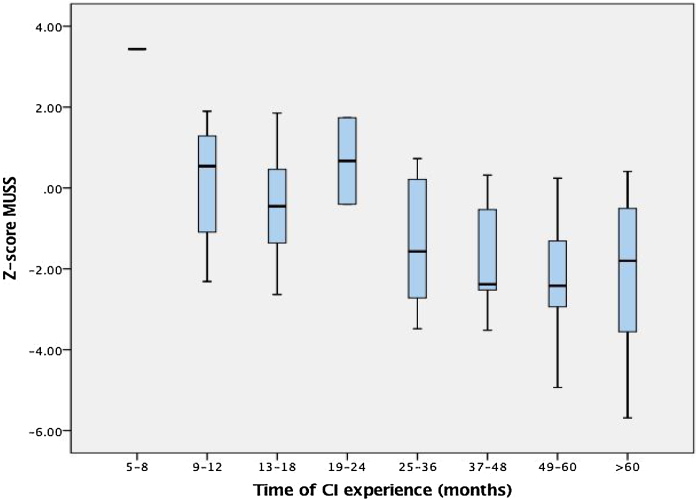
Sample performance on MUSS during different hearing ages (time of experience with Cochlear Implant [CI]) in median values with interquartile ranges and vertical bars which denote minimum and maximum values — outliers are depicted by circles outside the bars.

**Figure 3:**
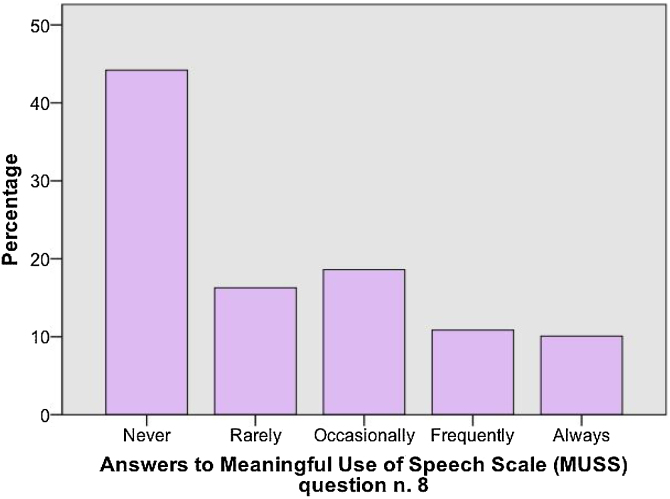
Sample performance on MUSS question number 8.

In relation to patient’s performance, auditory and speech skills scores for different times of hearing age (time of CI experience) in medians and interquartile ranges can be depicted in Figs. 1and 2. Inadequate results (below score-Z-1) for the MAIS score were found in 59.7% of the sample while for the MUSS score the proportion was of 62% of the sample. Particularly from the MUSS score, a subanalysis of answers for question number 8 was performed. That question evaluates how frequently other adults not familiar with them can understand the child’s speech. The proportion of parents who answered never, rarely, or occasionally was of 79.1% of patients, as demonstrated in Fig. 3. Mean ages at implantation for each age group of CI experience are demonstrated in Table 4. For both the scores there was an observed tendency of lower results according to growing age/CI experience, but without statistical significance (p = 0.65).

**Table 4:** Mean age at implantation for age groups of CI experience.

Age group of CI experience, mo	Mean (SD)	n
5‒8	32.0	1
9‒12	37.7 (26.7)	8
13‒18	37.4 (17.4)	7
19‒24	40.0 (1.4)	2
25‒36	30.9 (10.9)	15
37‒48	45.1 (26.2)	7
49‒60	38.2 (13.9)	15
>60	43.1 (16.2)	74
Total	40.5 (16.9)	129

## Discussion

This is the first report on pediatric cochlear implant outcomes of patients from a public health system program in Brazil with a significant number of participants that did not exclude patients according to etiologies or comorbidities. Therefore, we believe it provides a worthy perspective of results for this population in our country and, possibly, other developing nations.

There are, however, methodological aspects in this study that should be pointed, such as the use of subjective scales to measure language development, the self-reported nature of answers to many of the variables (such as IC usage, which would be better evaluated with datalogging information) and the retrospective nature of the analysis per se. Nevertheless, we consider that the exploratory nature of this study allows many valid inferences regarding our results and the variables possibly associated with them to be highlighted for further analysis in the future.

From the 189 implanted children, 31.7% were lost to follow-up during the 10 year interval time of the cochlear implant program which this study has focused on. This is an important group, yet to be studied, as their lack of progress in language skill development during the first few years following implantation may have contributed to dropping out of the study without communication or notification.

There was an overall lower score than expected both for auditory and for speech skills in our sample. The scores were compared to scores from a different sample, which excluded patients with comorbidities and additional disabilities.^40^ Even though there is an asymmetry between those samples, there aren’t studies with a more similar sample of participants in the national literature to compare our findings with.

Another reason we chose to show our results in the form of a comparison with a “gold-standard” sample is because the primary goal of childhood deafness treatment is to develop language skills as similar as possible to their peers with normal hearing. In that sense, the subanalysis of answers for question number 8 in the MUSS score shows only 21% of our sample has positive results on a straightforward question about their communication abilities in their daily lives. We considered those answers of particular interest since it evaluates how frequently other adults not familiar with the child can understand what they’re saying.

One possible explanation for those results is that the median age of implantation was higher than the sensitive period of 3.5 years. It is called sensitive because it’s the period for harnessing the height of cortical plasticity and for allowing auditory cortical maturation to progress appropriately, coinciding with the peak of synaptic density in auditory cortex, an indication of maximal plasticity.^15^, ^41^ More than 20 years ago, Nikolopoulos et al.^42^ showed important differences between children implanted early and late and, since then, strong evidence point to the importance of early diagnosis so that early treatment is also provided.^16^, ^43^, ^44^, ^45^, ^46^, ^47^

In Brazil, the deficient neonatal hearing screening program leads to late diagnosis and rehabilitation.^48^ National studies show great difficulty in reaching guideline’s recommended standards.^48^, ^49^ Brazilian studies found a mean age at diagnosis of 4.3 and 5.4 years and the mean age at the beginning of hearing aid use of 6.8 and 7.5 years.^50^, ^51^ In a previous study by our group,^52^ with a similar sample of patients, we found that the median age at the first appointment in a specialized centre and at the cochlear implant surgery was 1.4 (0.5–2.5) and 2.8 (1.8–4.6) years, respectively. Considering that neonatal hearing screening in Brazil has a national coverage rate of 34%,^48^ these numbers are comprehensible but reveal a big challenge for the public health administrators.

Another potential problem is the low rate of children with bilateral implants, mostly because it began to be carried out in Brazil only in 2014. The first issue is the privation of the clear benefits of bilateral hearing, which affects the domains of verbal perception of noise and the ability to localize the source of the noise.^10^ The second issue is that CI devices often present technical problems and need to be taken for technical maintenance. Bilateral implantation prevents these children from going back to auditory deprivation in these situations and could help to adhere to rehabilitation.

Regarding speech therapy, 69.8% of patients in our sample performed speech therapy in their city only and the approach used by local speech therapists was not evaluated. Growing evidence suggests that emphasis on Auditory-Verbal (AV) and Auditory-Oral (AO) communication is linked to better linguistic domains, higher levels of implant use, academic success, less communication breakdown and expressive language advantages.^53^ AV Therapy (AVT) is a structured program that fosters intense speech and language input to children with hearing impairment across different environmental settings by involving speech therapists, special educators, audiologists, and parents.^54^

In regard to speech therapy frequency, only 71.2% of our sample attended appointments regularly (at least once a week). In a study that compared two groups (High vs Low Language skills) of school-aged children who received CIs by 4 years of age it was demonstrated that the High Language group attended significantly more AV therapy appointments, which significantly predicted speech recognition performance in all testing conditions.^55^

In our sample, the number of programming sessions performed was, on average, 30% lower than the planned number. Added to that, more than 50% of the sample (72 patients) had to perform an audiometry during the study period because there weren’t any recent (since 2019) records of pure-tone thresholds in use of CI on their records. Although important, it is known that pure-tone thresholds don’t provide accurate information regarding the quality of acoustic information.^56^, ^57^ Ideally, the assessment of speech perception should ideally be carried out before the programming sessions, with the application of speech perception tests, both in noise and in silence, to verify if the devices are properly adapted and measure the benefits.^58^

A previous report by our group found that more than 60% of children treated for hearing loss in our tertiary centre are from the countryside.^52^ and live far from habilitation facilities, which is a factor that could explain the lack of adherence to AV therapy and programming sessions. This factor has been previously shown to have an inverse relationship with language scores.^14^

In this study, it was possible to observe homogeneity in relation to Socioeconomic Status (SES) and level of maternal education, but family’s motivation are a subject to be better understood since previous studies have linked qualitative aspects of communication between children and parents to positive variation in IQ, verbal comprehension, and vocabulary, even after adjusting for parental SES.^59^ As our CI program is part of a public health program, it is understandable that the families involved in the program belong to a lower socioeconomic status. Thus, it’s important to identify factors that enable these families to stimulate their children. In this context, encouraging activities such as reading habits, using language facilitation techniques and encouraging attendance at AV therapy seem to be the most fruitful way to achieve better results regardless of major changes in the structure of the public service.

However, it is important that more studies that evaluate the results of programs for the treatment of profound deafness in children draw attention to their characteristics so that there are well-established arguments that help change public policies. In this sense, evaluations of the factors involved in the outcomes presented in this paper will be carried out and published soon. Our mission, as health workers, is to provide reliable data about the reality with which we work and to suggest objective changes to seek better results.

## Conclusion

Our PEP strategy to evaluate results from our CI program revealed that patient’s profile is very similar in regard to SES, and they have an elevated mean age at cochlear implantation. In relation to engagement to postoperative treatment there is a high rate of loss to follow-up and low attendance to speech and programming sessions. Finally, there was a poor language performance (compared to clinical benchmark from another national study) in this pediatric CI program from the public health system in Southern Brazil.

## Funding

None to declare.

## Conflicts of interest

The authors declare no conflicts of interest.
